# Association between insomnia and frailty in older population: A meta‐analytic evaluation of the observational studies

**DOI:** 10.1002/brb3.2793

**Published:** 2022-12-14

**Authors:** Quan Wen, Xue Yan, Zhong Ren, Bo Wang, Yuqiu Liu, Xi Jin

**Affiliations:** ^1^ The third affiliated clinical hospital Changchun University of Chinese Medicine Changchun China; ^2^ Department of Cerebral Diseases The Affiliated Hospital of Changchun University of Chinese Medicine Changchun China; ^3^ Rehabilitation Center Nong'an County Hospital of Traditional Chinese Medicine Changchun China; ^4^ Physical Examination Center Rongda Hospital Changchun China

**Keywords:** cross‐sectional studies, frailty, insomnia, meta‐analysis, older people

## Abstract

**Introduction:**

Poor sleep quality has been suggested as a risk factor of frailty. However, previous studies that evaluated the association between insomnia and frailty in older population showed inconsistent results. We performed a meta‐analysis to comprehensively evaluate the association.

**Methods:**

Observational studies related to the aim of the meta‐analysis were identified by search of PubMed, Embase, and Web of Science databases. A random‐effect model incorporating the potential between‐study heterogeneity was used to pool the results.

**Results:**

Twelve studies including 16,895 old people contributed to the meta‐analysis. Pooled results suggested a significant association between insomnia and frailty in the older population (odds ratio [OR]: 1.95, 95% confidence interval [CI]: 1.52–2.41, *p* < .001; *I* ^2^ = 80%). Subgroup analyses showed consistent association between different symptoms of insomnia and frailty, including difficulty in falling asleep (OR: 1.45), difficulty in maintaining sleep (OR: 1.23), early morning awakening (OR: 1.21), and non‐restorative sleep (OR: 1.84, *p* for subgroup difference = .15). Results were also consistent for subgroup analyses according to the study country, sample size, cutoffs of age for defining the older population, proportions of men, diagnostic criteria for frailty, adjustment of depression, and scores of study quality (*p* for subgroup difference all > .05). However, a stronger association was observed for insomnia detected with the Athens Insomnia Scale (OR: 2.92) than that with Pittsburgh Sleep Quality Index (OR: 1.30) or self‐reporting (OR: 1.60, *p* for subgroup difference = .002).

**Conclusion:**

Insomnia is independently associated with frailty in the older population.

## Graphical abstract

A meta‐analysis of twelve studies showed that insomnia is independently associated with frailty in the older population. The association was consistent for different symptoms of insomnia, including difficulty in falling asleep, difficulty in maintaining sleep, early morning awakening, and non‐restorative sleep.

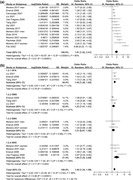

## 1 INTRODUCTION

Insomnia is a common sleep problem among the old people, with an overall prevalence reported between 30% and 50% (Dopheide, 2020; Patel, Steinberg, & Patel, 2018). The symptoms of insomnia include difficulty in falling asleep, difficulty in maintaining sleep, early morning awakening, and non‐restorative sleep (Ebben, 2021; Flaxer, Heyer, & Francois, 2021; Samara et al., 2020). Accumulating evidence suggests that people with insomnia are likely to have multiple somatic and psychologic disorders, impaired quality of life, and possibly higher all‐cause mortality (Cunnington, Junge, & Fernando, 2013; Ge et al., 2019; Javaheri & Redline, 2017). In the older people, untreated insomnia has been related to various comorbidities, such as anxiety and depression, cognitive impairment, diabetes and metabolic syndrome, hypertension, stroke, and coronary artery diseases. (Patel et al., 2018). Moreover, the presence of insomnia contributes significantly to the development of work disability, sick leave, and poor performance at work (Kucharczyk, Morgan, & Hall, 2012), all of which significantly impair the functional capacity and quality of life of the affected population (Abad & Guilleminault, 2018; Gillam, 2009).

Frailty is a common geriatric syndrome which is defined as a state of age‐related impairment of biological reserve, reduction of the ability to maintain physiological balance and increment of the vulnerability to adverse outcomes (Lee, Lee, & Jang, 2020; Rohrmann, 2020). In the older population, frailty has been also related to an increased risk of cognitive decline, poor prognosis of various clinical conditions, and a higher risk of all‐cause mortality (Pilotto et al., 2020; Vermeiren et al., 2016). There is evidence that insomnia causes dysfunctional sleep, fatigue, unsteady gait, and physical inactivity, which all increase frailty risk (Wai & Yu, 2020). Moreover, untreated insomnia has been related to various psychological and somatic disorders, which may also increase the vulnerability to frailty (Cochen et al., 2009). Accordingly, it has been proposed that insomnia may be an associated factor of frailty (Cochen et al., 2009). However, previous studies evaluating the possible association between insomnia and frailty showed inconsistent results (Cavusoglu et al., 2021; Devkota, Anderson, Soiza, & Myint, 2017; Ensrud et al., 2009; Fan et al., 2022; Liu et al., 2021; Moreno‐Tamayo, Manrique‐Espinoza, Guerrero‐Zuniga, Ramirez‐Garcia, & Sanchez‐Garcia, 2021; Moreno‐Tamayo, Manrique‐Espinoza, Rosas‐Carrasco, Perez‐Moreno, & Salinas‐Rodriguez, 2017; Pacholek et al., 2020; Tang et al., 2021; Vaz Fragoso, Gahbauer, Van Ness, & Gill, 2009; Xie, Ma, Chen, & Wang, 2021; Zhao, Gao, Li, & Wang, 2019), and it has also not determined if the difference in the symptoms of insomnia may affect the potential association. Accordingly, a systematic review and meta‐analysis was performed to explore the association between insomnia and frailty in the older population.

## 2 MATERIALS AND METHODS

The preferred reporting items for systematic reviews and meta‐analyses (PRISMA) statement (Page, McKenzie, et al., 2021; Page, Moher, et al., 2021) and the Cochrane's handbook (Higgins et al., 2021) guideline were followed in the conceiving, conducting, and reporting the study.

### 2.1 Search of databases

Studies were retrieved by search of the electronic databases including PubMed, Embase, and Web of Science database to February 15, 2022, with a combined search term as (“insomnia” OR “sleep”) AND (“frailty” OR “frail”). The search was restricted to human studies with no limitation of the publication language. The reference lists of the relevant original and review articles were also manually screened for possible related studies. Only studies published as full‐length articles in peer‐reviewed journals were included in the meta‐analysis.

### 2.2 Study inclusion and exclusion criteria

The inclusion criteria were developed according to the aim of the meta‐analysis, with the recommended PICOS criteria.

P (patients): Older people (60 years old or above) living in communities or long‐term care facilities.
I (exposure): Patients with insomnia.
C (control): Patients without insomnia. The definition and diagnostic criteria for insomnia were in accordance with the criteria applied in the original studies.
O (outcomes): Frailty as diagnosed by the criteria used in the original studies.
S (study design): Observational studies.

Reviews, editorials, meta‐analyses, studies including non‐older population, or studies that did not analyze insomnia or report frailty were excluded. For studies with overlapped population, the one with the largest sample size was included.

### 2.3 Data collection and quality assessing

The literature search, data collection, and study quality assessment were independently conducted by two authors separately. If discrepancies occurred, the corresponding author was contacted for discussion to reach the consensus. We collected data regarding study information, participant characteristics, methods for the diagnosis of insomnia, number of older people with insomnia, methods for the diagnosis of frailty, number of older people with frailty, and variables adjusted when the association between insomnia and frailty was presented. Study quality was assessed via the Newcastle–Ottawa Scale (Wells et al., 2010) with scoring regarding the criteria for participant selection, comparability of the groups, and the validity of the outcomes. The scale ranged between 1 and 9 stars, with larger number of stars presenting higher study quality.

### 2.4 Statistical analyses

The association between insomnia and frailty in the older population was presented as odds ratio (OR) and the 95% confidence interval (CI). For studies that analyzed the above association with multiple models, data with the most adequately adjusted model were extracted for the meta‐analysis. Using the data of 95% CIs or *p* values, data of ORs and the standard errors (SEs) could be calculated, and a subsequent logarithmical transformation was conducted to keep stabilized variance and normalized distribution. Between study heterogeneity was estimated with the Cochrane's Q test and the *I* ^2^ statistic (Higgins & Thompson, 2002), with *I* ^2^ >50% reflecting the significant heterogeneity. A random‐effect model was applied to combine the results by incorporating the influence of heterogeneity (Higgins et al., 2021). Sensitivity analysis by excluding one dataset at a time was performed to evaluate the influence of individual study on the results of the meta‐analysis (Patsopoulos, Evangelou, & Ioannidis, 2008). Subgroup analyses were performed to evaluate the influence of study characteristics on the outcome, such as different symptoms of insomnia, study country, sample size, cutoffs of age for defining the older population, proportions of men, methods for the diagnosis of insomnia, diagnostic criteria for frailty, adjustment of depression, and scores of study quality. Medians of the continuous variables were used to define subgroups. In addition, univariate meta‐regression analysis was also performed to evaluate if the difference in study characteristics of continuous variables may affect the association, such as sample size, mean age, prevalence of insomnia, prevalence of frailty, and quality scores. By construction of the funnel plots, the publication bias was estimated based on the visual judgement of the symmetry of the plots, supplemented with the Egger's regression asymmetry test (Egger, Davey Smith, Schneider, & Minder, 1997). The RevMan (version 5.1; Cochrane Collaboration, Oxford, UK) and Stata software (version 12.0; Stata Corporation, College Station, TX, USA) were applied for these analyses.

## 3 RESULTS

### 3.1 Literature search

**FIGURE 1:**
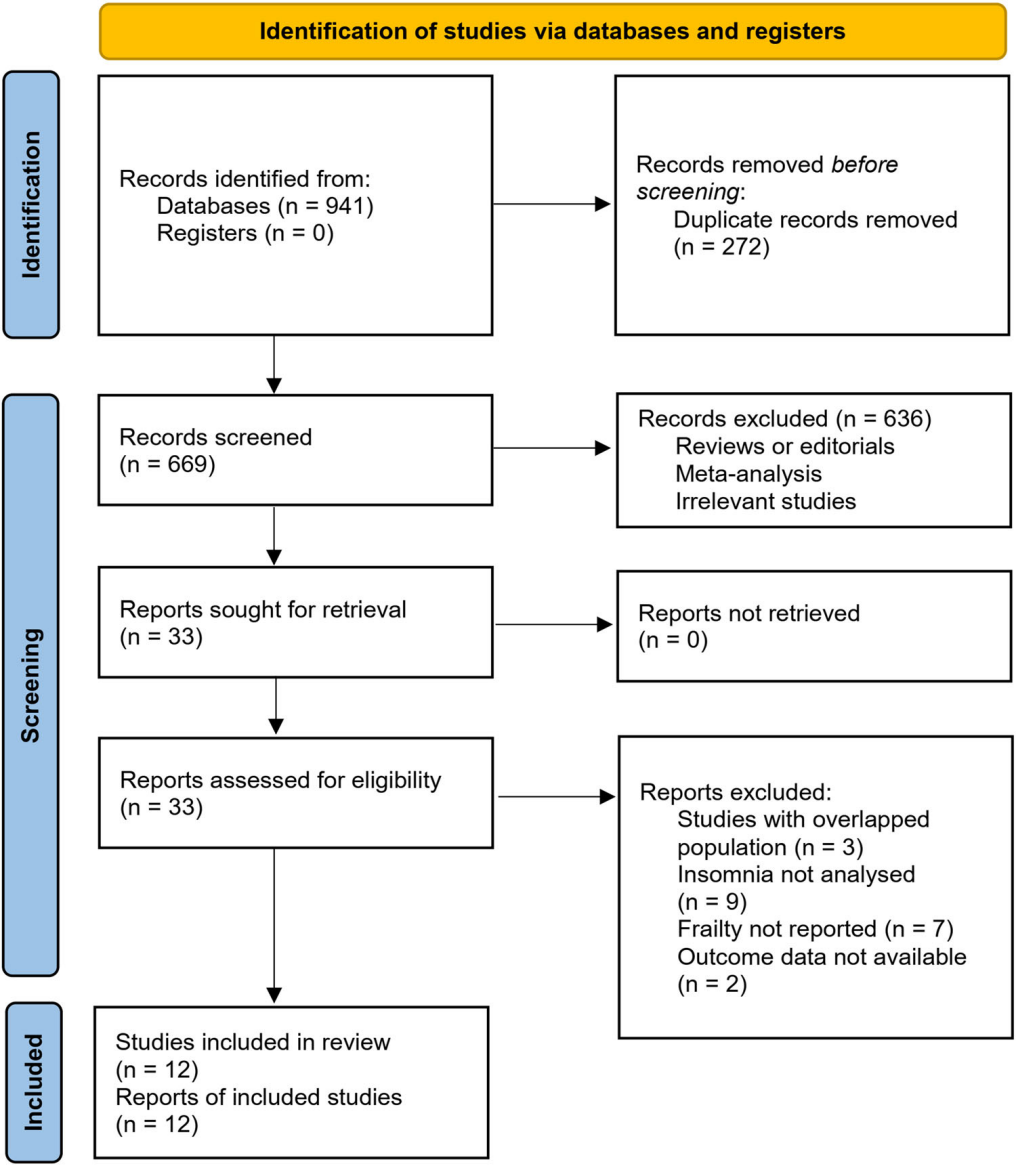
Diagram of database search and study inclusion

The flowchart of literature search and study inclusion is displayed in Figure 1. In summary, 941 records were obtained in the initial database search, and 272 were removed for duplications. Subsequently, 669 studies were screened with titles and abstracts, and 636 were excluded largely because they were not relevant to the objective of the meta‐analysis. Finally, 33 studies underwent full‐text review, and 21 were excluded for the reasons listed in Figure 1, which eventually made 12 studies available for the meta‐analysis.

### 3.2 Study characteristics

Overall, 12 cross‐sectional studies (Cavusoglu et al., 2021; Devkota et al., 2017; Ensrud et al., 2009; Fan et al., 2022; Liu et al., 2021; Moreno‐Tamayo et al., 2021; Moreno‐Tamayo et al., 2017; Pacholek et al., 2020; Tang et al., 2021; Vaz Fragoso et al., 2009; Xie et al., 2021; Zhao et al., 2019) including 16,895 old people contributed to the meta‐analysis. The characteristics of the included studies are summarized in Table 1. The studies were published between 2009 and 2021 and were performed in the United States (Ensrud et al., 2009; Liu et al., 2021; Vaz Fragoso et al., 2009), Nepal (Devkota et al., 2017), Poland (Pacholek et al., 2020), China (Fan et al., 2022; Tang et al., 2021; Xie et al., 2021; Zhao et al., 2019), Mexico (Moreno‐Tamayo et al., 2021; Moreno‐Tamayo et al., 2017), and Turkey (Cavusoglu et al., 2021). All the studies included older people aged at least 60 years from the communities (Cavusoglu et al., 2021; Devkota et al., 2017; Ensrud et al., 2009; Fan et al., 2022; Liu et al., 2021; Moreno‐Tamayo et al., 2021; Moreno‐Tamayo et al., 2017; Pacholek et al., 2020; Tang et al., 2021; Vaz Fragoso et al., 2009; Xie et al., 2021) or nursing homes (Zhao et al., 2019). Diagnosis of insomnia was according to the Insomnia Severity Index (ISI) (Vaz Fragoso et al., 2009), Pittsburgh Sleep Quality Index (Cavusoglu et al., 2021; Ensrud et al., 2009; Tang et al., 2021), the Athens Insomnia Scale (AIS) (Fan et al., 2022; Moreno‐Tamayo et al., 2021; Pacholek et al., 2020; Zhao et al., 2019), or self‐reported symptoms of insomnia (Devkota et al., 2017; Liu et al., 2021; Moreno‐Tamayo et al., 2017; Xie et al., 2021), and the overall prevalence of insomnia was 6105/16895 (36.1%). Identification of frailty was mostly achieved with the Fried Frailty Phenotype tool (Cavusoglu et al., 2021; Liu et al., 2021; Moreno‐Tamayo et al., 2021; Moreno‐Tamayo et al., 2017; Vaz Fragoso et al., 2009; Xie et al., 2021), and also with instruments such as Cardiovascular Health Study frailty index (Ensrud et al., 2009), the Canadian Study of Health and Aging frailty tool (Devkota et al., 2017), the FRAIL scale (Tang et al., 2021; Zhao et al., 2019), the Clinical Frailty Scale (Pacholek et al., 2020), and the Tilburg Frailty Indicator (Fan et al., 2022). The overall prevalence of frailty was 2804/16895 (16.6%). Possible confounding factors such as age, sex, education, living status, daily activities, comorbidities, and concomitant medications were adjusted of a varying degree in the included studies when the association between insomnia and frailty was presented in multivariate analyses. The Newcastle–Ottawa Scale (NOS) of the included studies were all seven to nine stars, suggesting generally good study quality (Table 2).

**TABLE 1:** Characteristics of the included studies

Study	Country	Design	Participant characteristics	Sample size	Mean age (years)	Men (%)	Diagnosis of insomnia	No. of people with insomnia	Diagnosis of frailty	No. of people with frailty	Variables adjusted
Vaz Fragoso et al. (2009)	USA	CS	Community‐living old people ≥75 years	374	84.3	32.6	ISI	160	FFP	154	Age, sex, cognitive impairment, comorbidities, depressive symptoms, and concomitant medications
Ensrud et al. (2009)	USA	CS	Community‐derived old men ≥67 years	3133	76.4	100	PSQI	1383	CHS frailty index	437	Age, race, site, health status, educational level, social support, alcohol intake, smoking status, depressive symptoms, cognitive function, functional disabilities, BMI, and concomitant medications
Devkota et al. (2017)	Nepal	CS	Community‐derived old people ≥60 years	253	79.1	32	Self‐reported	123	CSHA frailty tool	70	Age, sex, smoking, living arrangements, and comorbidities
Moreno et al. (2017)	Mexico	CS	Community‐living old people ≥60 years	591	76.3	47.2	Self‐reported	118	FFP	63	Age, sex, ethnicity, education, cognitive status, daily activity, physical performance, and concomitant medications
Zhao et al. (2019)	China	CS	Older adults (≥60 years) in nursing homes	370	77.6	40.5	AIS	169	FRAIL scale	108	Age, sex, ethnicity, education, depression, and daily activities
Pacholek et al. (2020)	Poland	CS	Community‐derived old people ≥65 years	438	75.6	37	AIS	182	CFS	144	Age, sex, living condition, daily activity, cognitive status, and concomitant medications
Xie et al. (2021)	China	CS	Community‐living old people ≥75 years	1585	81.4	41.8	Self‐reported	731	FFP	225	Age, sex, education, marital status, depression, comorbidity, and concomitant medications
Liu et al. (2021)	USA	CS	Community‐derived old people ≥65 years	7609	74.2	43.5	Self‐reported	2278	FFP	1065	Age, sex, pain, and depression
Moreno‐Tamayo et al. (2021)	Mexico	CS	Community‐living old people ≥60 years	1643	67.1	50.1	AIS	497	FFP	172	Age, sex, education, married/civil union, current alcohol consumption, current smoking, clinically significant depression symptoms, cognitive decline, comorbidities, and concomitant medications
Tang et al. (2021)	China	CS	Community‐living old people ≥65 years	345	80.3	20	PSQI	195	FRAIL scale	140	Age and sex
Cavusoglu et al. (2021)	Turkey	CS	Community‐derived old people ≥80 years	100	83.5	45	PSQI	58	FFP	41	Age, sex, daily activity, and comorbidities
Fan et al. (2022)	China	CS	Community‐living old people ≥60 years	454	69.1	42.3	AIS	211	TFI	185	Age, sex, marital status, residence, monthly personal income, level of education and comorbidity

Abbreviations: BMI, body mass index; CS, cross‐sectional; CFS, Clinical Frailty Scale; ISI, Insomnia Severity Index; FFP, fried frailty phenotype; PSQI, Pittsburgh Sleep Quality Index; CHS, Cardiovascular Health Study; CSHA, Canadian Study of Health and Aging; AIS, Athens Insomnia Scale; TFI, Tilburg Frailty Indicator.

**TABLE 2:** Details of study quality evaluation via the Newcastle‐Ottawa Scale

Study	Adequate definition of cases	Representativeness of cases	Selection of controls	Definition of controls	Control for age and sex	Control for other confounders	Exposure ascertainment	Same methods for events ascertainment	Non‐response rates	Total
Vaz Fragoso et al. (2009)	1	1	1	1	1	1	1	1	1	9
Ensrud et al. (2009)	1	1	1	1	1	1	1	1	1	9
Devkota et al. (2017)	1	1	1	1	1	0	0	1	1	7
Moreno‐Tamayo et al. (2017)	1	1	1	1	1	0	0	1	1	7
Zhao et al. (2019)	1	1	1	1	1	0	1	1	1	8
Pacholek et al. (2020)	1	1	1	1	1	0	1	1	1	8
Xie et al. (2021)	1	1	1	1	1	1	0	1	1	8
Liu et al. (2021)	1	1	1	1	1	0	0	1	1	7
Moreno‐ Tamayo et al. (2021)	1	1	1	1	1	1	1	1	1	9
Tang et al. (2021)	1	1	1	1	1	0	1	1	1	8
Cavusoglu et al. (2021)	1	1	1	1	1	0	1	1	1	8
Fan et al. (2022)	1	1	1	1	1	0	1	1	1	8

### 3.3 Meta‐analysis results

**FIGURE 2:**
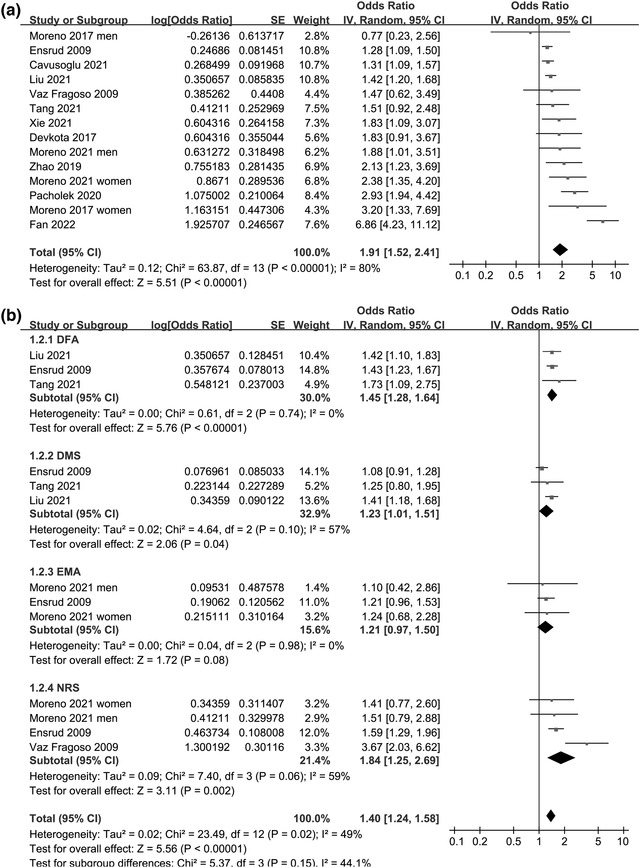
Forest plots for the meta‐analyses regarding the association between insomnia and frailty in the older population: (a) overall meta‐analysis and (b) subgroup analysis according to the symptoms of insomnia. Abbreviations: CI, confidence interval; DFA, difficulty in falling asleep; DMS, difficulty in maintaining sleep; EMA, early morning awakening; NRS, non‐restorative sleep; OR, odds ratio

Two studies reported the outcome by sex (Moreno‐Tamayo et al., 2021; Moreno‐Tamayo et al., 2017), which were included as independent datasets in the meta‐analysis. Accordingly, 14 datasets from 12 studies were available for the overall meta‐analysis. Pooled results suggested a significant association between insomnia and frailty in the older population (OR: 1.95, 95% CI: 1.52–2.41, *p* < .001; *I* ^2^ = 80%; Figure 2a). Sensitivity analyses by excluding one dataset at a time did not significantly change the result (OR: 1.65–2.01, *p* all < .05). Subgroup analyses showed consistent association between different symptoms of insomnia and frailty, including difficulty in falling asleep (OR: 1.45, 95% CI: 1.28–1.64, *p* < .001), difficulty in maintaining sleep (OR: 1.23, 95% CI: 1.01–1.51, *p* = .04), early morning awakening (OR: 1.21, 95% CI: 0.97–1.50, *p* = .08), and non‐restorative sleep (OR: 1.84, 95% CI: 1.25–2.69, *p* = .002; *p* for subgroup difference = .15; Figure 2b). Results were also consistent for subgroup analyses according to the study country, sample size, cutoffs of age for defining the older population, proportions of men, diagnostic criteria for insomnia, evaluating tools for frailty, adjustment of depression, and scores of study quality (*p* for subgroup effect all < .05; Table 3). However, a stronger association was observed for insomnia detected with the AIS (OR: 2.92) than that with the PSQI (OR: 1.30) or self‐reported symptoms of insomnia (OR: 1.60, *p* for subgroup difference = .002). Further meta‐regression analyses did not support that differences in study characteristics of continuous variables may have a significant influence on the association between insomnia and frailty (*p* all > .05; Table 4).

**TABLE 3:** Results of subgroup analyses

Study characteristics	Datasets number	OR (95% CI)	*I* ^2^	*p* For subgroup effect	*p* For subgroup difference
Country					
Asian	4	2.45 [1.16, 5.15]	87%	.02	
Non‐Asian	10	1.66 [1.37, 2.01]	63%	<.001	.32
Sample size					
<500	7	2.19 [1.36, 3.54]	87%	.001	
≥500	7	1.54 [1.27, 1.88]	44%	<.001	.18
Age cutoffs					
<65 years	7	2.44 [1.51, 3.94]	73%	.003	
≥65 years	7	1.52 [1.27, 1.80]	61%	<.001	.07
Men					
<40%	6	2.17 [1.67, 2.83]	16%	<.001	
≥40%	8	1.79 [1.34, 2.39]	85%	<.001	.33
Diagnosis of insomnia					
PSQI	3	1.30 [1.16, 1.46]	0%	<.001	
AIS	5	2.92 [1.84, 4.61]	74%	<.001	
Self‐reported	5	1.60 [1.23, 2.08]	25%	<.001	.002
Diagnosis of frailty					
FFP	8	1.53 [1.29, 1.81]	29%	<.001	
Others	6	2.29 [1.34, 3.92]	90%	.002	.16
Depression adjusted					
Yes	7	1.51 [1.29, 1.77]	30%	<.001	
No	7	2.19 [1.29, 3.73]	88%	.004	.19
NOS					
7	4	1.59 [1.09, 2.33]	36%	.02	
8	6	2.29 [1.38, 3.80]	89%	.001	
9	4	1.59 [1.15, 2.19]	44%	.005	.44

Abbreviations: AIS, Athens Insomnia Scale; CI, confidence interval; FFP, fried frailty phenotype; OR, odds ratio; NOS, the Newcastle–Ottawa Scale; PSQI, Pittsburgh Sleep Quality Index.

**TABLE 4:** Univariate meta‐regression analysis

Covariate	Coefficient	95% CI	*p*
Sample size	−0.000065	−0.000181 to 0.000052	.277
Mean age (years)	−0.059	−0.172 to 0.054	.361
Male (%)	−0.0056	−0.0183 to 0.0070	.391
Prevalence of insomnia (%)	−0.0028	−0.0313 to 0.0257	.848
Prevalence of frailty (%)	0.012	−0.010 to 0.034	.296
Quality score	−0.040	−0.463 to 0.383	.853

Abbreviation: CI, confidence interval.

### 3.4 Publication bias

**FIGURE 3:**
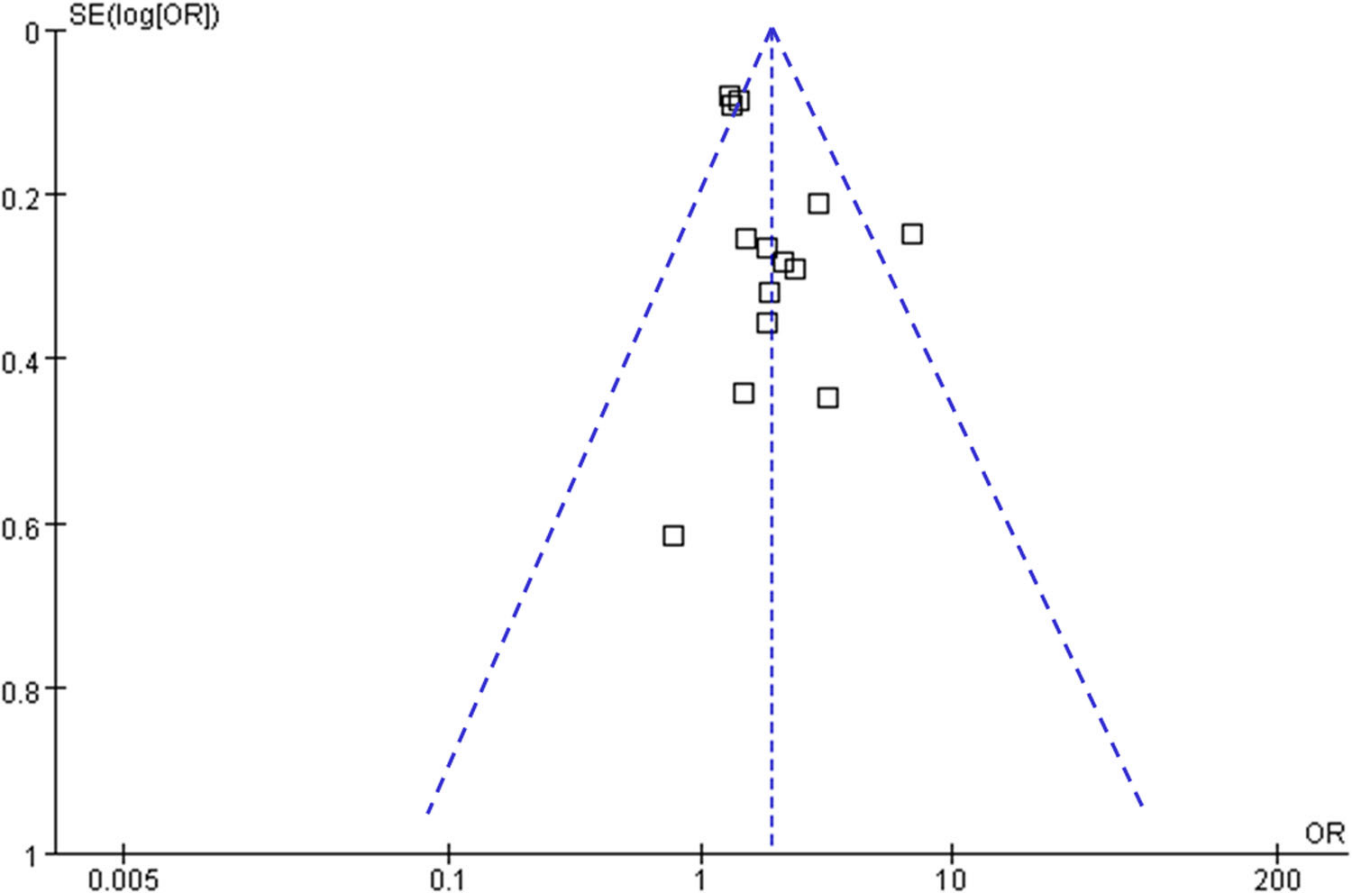
Funnel plots for the publication bias underlying the meta‐analysis regarding the association between insomnia and frailty in the older population

Figure 3 displays the funnel plots for the association between insomnia and frailty. Visual inspection revealed symmetry of the plots, reflecting a low risk of publication biases. The Egger's regression tests also indicated low risk of publication biases (*p* = .23).

## 4 DISCUSSION

In this study, by pooling the results of 12 cross‐sectionals studies, we found that insomnia is independently associated with frailty in older people from communities or nursing homes. Subgroup analyses showed consistent association for different symptoms of insomnia. Moreover, further subgroup analyses confirmed that the association was consistent and was not significantly affected by study characteristics such as the study country, sample size, cutoffs of age for defining the older population, proportions of men, diagnostic criteria for frailty, adjustment of depression, and scores of study quality. Consistently, meta‐regression analysis also did not support that difference in study characteristics of continuous variables such as sample size, mean age, prevalence of insomnia, prevalence of frailty, and quality scores may significantly affect the association between insomnia and frailty. However, a stronger association between insomnia and frailty was observed for insomnia detected with the AIS than that with the PSQI or self‐reported symptoms of insomnia. Taken together, these results suggest that insomnia is independently associated with frailty in the older population.

The strengths of the meta‐analysis include extensive literature search, including studies with multivariate analyses, and comprehensive evaluation with multiple sensitivity and subgroup analyses. Specifically, we found that insomnia defined according to the different symptoms is all consistently associated with frailty in the older population, reflecting a universal relationship between insomnia and frailty in this population. Moreover, due to the fact that both insomnia and frailty are likely to be associated with multiple comorbidities, particularly in the older, it is important to determine whether the association between insomnia and frailty is independent of possible confounding factors. All of the included studies used multivariate analyses to determine the association between insomnia and frailty, at least for age and sex. Because depression has been related to both insomnia and frailty (Baglioni et al., 2011; Soysal et al., 2017), it could be hypothesized that depression may be an important confounding factor for the association between insomnia and frailty. Our subgroup showed consistent results for the association between insomnia and frailty in studies with and without the adjustment of frailty, which further confirmed the robustness of the association that may not be affected by depressive symptoms of the people. In addition, although all the subgroup analyses consistently showed a significant association between insomnia and frailty, we observed a stronger association in studies with insomnia evaluated with the AIS compared to those with PSQI and self‐reported symptoms. The reasons for the finding are currently unknown. Although both the AIS and PSQI are validated tools for the evaluation of the sleep quality, consensus has not been reached regarding the optimal tools and cutoffs for the identification of poor sleepers in the older (Fabbri et al., 2021). Future studies are warranted.

The pathophysiological basis for the association between insomnia and frailty in the older people remains not fully understood. It has been suggested that poor sleep quality may lead to a series of changes in hormonal, metabolic, and inflammatory status of the people. For example, insomnia has been related with insulin resistance, activated inflammatory response, and enhanced systematic oxidative stress (Hashemipour, Ghorbani, Khashayar, & Olfati, 2021; Irwin & Vitiello, 2019; Kim & Yoon, 2020), all of which may lead to protein synthesis impairment and sarcopenia, a major cause of physical frailty in the older (Cleasby, Jamieson, & Atherton, 2016; Umegaki, 2016). Studies are needed for further clarification of the mechanisms.

The clinical implications of the results of the meta‐analysis should also be noticed. Currently, sleep disorders are not screened and treated in the majority of health care services for frail older adults. Due to the importance of frailty in current geriatric medicine, it is important to perform a comprehensive geriatric evaluation in older patients with insomnia. On the other hand, it may be more important to evaluate whether interventions to improve sleep quality of the older people with insomnia could improve the frailty outcomes as well. However, to the best of our knowledge, no study has been conducted to directly observe the influence of insomnia treatment on frailty outcome in the older population. In addition to pharmacological interventions, there are several non‐pharmacological interventions for promoting sleep quality with minimal side effects and recognized benefits, such as cognitive behavior therapy and brief behavioral treatment (Chan, Chan, Li, & Wing, 2021). Studies are urgently needed in this regard.

The meta‐analysis also has several limitations. First, all of the included studies were of cross‐sectional designs. Accordingly, it could not be determined whether insomnia is an independent risk factor for frailty. Prospective cohort studies are needed for subsequent investigation. In addition, as a meta‐analysis of observational studies, the observed association between insomnia and frailty may also be confounded by some residual factors despite using the multivariate analyses among the included studies. Finally, the instruments used for diagnosis of frailty varied among the included studies, which may lead to between‐study heterogeneity. However, the optimal instrument for the evaluation of frailty in the older population remains to be established (Church, Rogers, Rockwood, & Theou, 2020). The fried frailty phenotype (FFP) was used in six of the included studies for determination of frailty, and subgroup analysis showed no significant difference in the association between studies using FFP and those with the other instruments.

## 5 CONCLUSION

In conclusion, results of this meta‐analytic study suggest that insomnia is independently associated with frailty in the older population. Prospective studies are needed to determine whether insomnia is an independent risk factor for frailty, and clinical studies should be considered to assess whether interventions targeting insomnia could improve the frailty outcomes in the older population.

## AUTHOR CONTRIBUTIONS

Quan Wen and Xi Jin conceived the study. Quan Wen and Xue Yan performed literature search, data extraction, and study quality evaluation. Zhong Ren, Bo Wang, and Yuqiu Liu performed statistical analyses and interpreted the results. Quan Wen drafted the manuscript. Xi Jin revised the manuscript. All author approved the submission of the manuscript.

## CONFLICT OF INTEREST

The authors declare no conflict of interest.

### PEER REVIEW

The peer review history for this article is available at https://publons.com/publon/10.1002/brb3.2793.

## Data Availability

All data generated or analyzed during this study are included in this article. Further enquiries can be directed to the corresponding author.
